# Reliability of the assessment of non-technical skills by using video-recorded trauma resuscitations

**DOI:** 10.1007/s00068-020-01401-5

**Published:** 2020-07-02

**Authors:** Oscar E. C. van Maarseveen, Wietske H. W. Ham, Roel L. N. Huijsmans, Rianne G. F. Dolmans, Luke P. H. Leenen

**Affiliations:** 1 Department of Trauma Surgery, University Medical Center Utrecht, Heidelberglaan 100, 3584 CX Utrecht, The Netherlands; 2 Institute of Nursing Studies, University of Applied Sciences Utrecht, Heidelberglaan 7, 3584 CS Utrecht, The Netherlands

**Keywords:** Non-technical skills, T-NOTECHS, Video analysis, Trauma team, Assessment

## Abstract

**Purpose:**

Non-technical skills have gained attention, since enhancement of these skills is presumed to improve the process of trauma resuscitation. However, the reliability of assessing non-technical skills is underexposed, especially when using video analysis. Therefore, our primary aim was to assess the reliability of the Trauma Non-Technical Skills (T-NOTECHS) tool by video analysis. Secondarily, we investigated to what extent reliability increased when the T-NOTECHS was assessed by three assessors [average intra-class correlation (ICC)] instead of one (individual ICC).

**Methods:**

As calculated by a pre-study power analysis, 18 videos were reviewed by three research assistants using the T-NOTECHS tool. Average and individual degree of agreement of the assessors was calculated using a two-way mixed model ICC.

**Results:**

Average ICC was ‘excellent’ for the overall score and all five domains. Individual ICC was classified as ‘excellent’ for the overall score. Of the five domains, only one was classified as ‘excellent’, two as ‘good’ and two were even only ‘fair’.

**Conclusions:**

Assessment of non-technical skills using the T-NOTECHS is reliable using video analysis and has an excellent reliability for the overall T-NOTECHS score. Assessment by three raters further improve the reliability, resulting in an excellent reliability for all individual domains.

## Introduction

The introduction of trauma teams has led to improved management and outcomes of severely injured patients [1–3]. A trauma team is a multidisciplinary group of health-care workers who collectively work together on the initial assessment and treatment of severely injured patients [4]. In this context, optimal technical performance of interventions is emphasized in resuscitation guidelines [5]. However, coordinated performance of such interventions within trauma teams requires more than mastering technical skills. Non-technical skills such as task management, leadership, situational awareness, communication and decision-making could be defined as cognitive, behavioral and social skills that contribute to safe and efficient team performance [6–10].

As the added value of non-technical skill training on patient safety, process efficiency and medical errors is shown by a growing number of studies [6–17], the issue of assessment becomes increasingly relevant. Therefore, there is a demand for a simple, validated and reliable assessment tool to lower the threshold for trauma centers to incorporate such assessments in their quality audits.

The T-NOTECHS is a tool developed to assess non-technical skills of the trauma team during trauma resuscitation [18]. The T-NOTECHS, stands for Trauma NOn-TECHnical Skills and is based on the NOTECHS, which was initially used to assess non-technical skills in aviation [19] and later on adapted and applied to assess non-technical skill performance of surgical teams [20]. As described by Steinemann et al. [18], the T-NOTECHS was developed by a panel of trauma practitioners composed of two trauma surgeons, one trauma/medical intensivist, and two critical care nurses. The T-NOTECHS consists of five behavioral domains: leadership, cooperation and resource management, communication and interaction, assessment and decision making, and situation awareness/coping with stress [18].

The T-NOTECHS is, to our opinion, a simple and validated instrument, but the reliability as found by Steinemann et al. [18] was low (ICC 0.48). An ICC of 0.48 means that 48% of the observed variance in T-NOTECHS scores is due to systematic differences compared to the total variance in achievement scores [21]. These values are especially low when aiming to assess the impact of training on non-technical skills over time.

To our knowledge, the reliability of the T-NOTECHS has only been tested during actual resuscitations by real-time observers and not by video analysis [18]. Video recordings particularly provide an indisputable, unbiased and accurate documentation of complex events and could therefore improve the reliability of the T-NOTECHS. Furthermore, video allows to assess the same resuscitation by multiple assessors, without interfering with the resuscitation process. In this study, the primary aim was to assess the reliability of the T-NOTECHS tool by assessing non-technical skills of trauma team with video analysis during actual trauma resuscitation. Secondarily, we investigated to what extent reliability increased in case T-NOTECHS was assessed by three assessors (average ICC) instead of one (individual ICC).

## Methods

### Design and sample

We retrospectively analyzed videos of consecutive trauma resuscitations. The trauma team was assessed on non-technical skills using the T-NOTECHS tool. To measure the interobserver reliability (a fully crossed design was used), all included videos were reviewed by all three assessors independently.

### Setting

This study took place in a level one trauma center in the Netherlands. Conform institutional's protocol, the trauma team is activated in case of (potentially) severely injured patient, which is predefined by physiological or anatomical criteria or mechanism of trauma was applicable. The trauma team, at our institution, consists basically of a trauma team leader, a surgical resident under direct supervision of a trauma surgeon, an anesthetist, one or two emergency department (ED) nurses, and a radiology technician. There are no differences in trauma team composition during the night or day. The tasks of each team member are in described in detail by Kreb et al. [22].

### Data collection

**Fig. 1:**
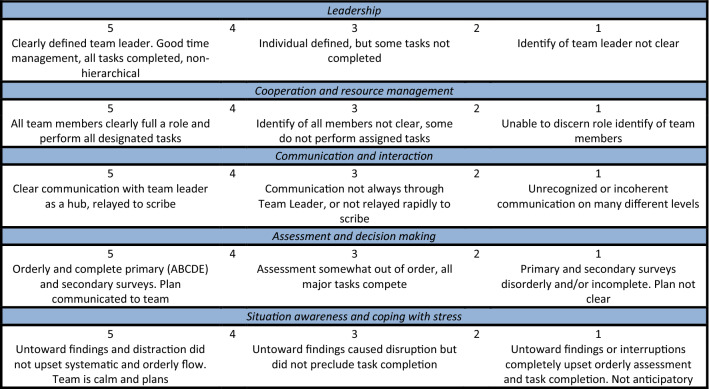
T-NOTECHS assessment tool [14]

As part of our standard quality audit, all trauma resuscitations by a trauma team are recorded on video prospectively. Eighteen recorded videos of trauma resuscitations were used to analyze non-technical skills of the trauma team. The baseline characteristics of resuscitated patients were collected. Three trained research assistants analyzed the recorded videos, who were respectively fourth (two of the three) and sixth (one of the three) year medical students. Before the analysis of the recorded videos using T-NOTECHS, the research assistants had 1 year experience with analyzing trauma resuscitations, while they had already been trained and gained experience in video analysis of Advanced Trauma Life Support (ATLS) adherence during resuscitation of trauma patients. Furthermore, prior to the assessment of the 18 videos, training sessions were yielded to align assessments of non-technical skills of the research assistants. The training consisted of reading the article of Steinemann et al. [18] and a 2 h training session where assessment of resuscitations using the T-NOTECHS tool was discussed. The research assistants were blinded to each other’s results. Videos were assessed on a computer inside the hospital building using a standardized score sheet in Microsoft Excel (Microsoft Corp. Released 2007. Microsoft Office Excel 2007, Version 12.0. Redmond, WA: Microsoft Corp.). All five behavioral domains of the T-NOTECHS were scored on a five-point Likert scale following the guidelines as described by Steinemann et al. [18] (Fig. 1). Five points indicate perfect behavior in a behavioral domain and one point indicates the team did not demonstrate this behavior. The sum of the scores of each behavioral domain ranged from 5 to 24, and a total of 25 points indicates perfect teamwork and a total of 5 points indicates ineffective teamwork.

### Sample size calculation

We performed a pre-study power analysis by using the formula proposed by Walter et al. [23]. The ICC we expected (*ρ*1) was 0.85 and the lowest ICC we would accept (*ρ*0) was 0.6. We had three raters (*n*) to assess all videos. In our sample size calculation, the alpha (*α*) level and (1 − *β*) was set at 0.05 and 0.80, respectively. Finally, we used a dropout rate of 15%, in case technical issues would appear. We used Microsoft Excel (Microsoft Corp. Released 2007. Microsoft Office Excel 2007, Version 12.0. Redmond, WA: Microsoft Corp.) to calculate the needed sample size. According to our sample size analysis, we needed to assess 18 videos of trauma resuscitations.

### Statistical analysis

Reliability is defined as the extent to which measurements can be replicated. In other words, it reflects not only the degree of correlation, but also agreement between measurements [24]. To assess interobserver reliability, the intra-class correlation (ICC) and corresponding 95% confidence interval (CI) weres calculated using SPSS (IBM Corp. Released 2012. IBM SPSS Statistics for Windows, Version 21.0. Armonk, NY: IBM Corp.). Higher ICC values indicate a greater degree of agreement between raters. An ICC estimate of 1 indicates perfect agreement and 0 indicates only random agreement. Negative ICC estimates indicate systematic disagreement [25]. In this study, we used cutoffs according to Cicchetti et al. [26] for qualitative ratings of agreement based on ICC values, with interobserver reliability being poor for ICC values less than 0.40, fair for values between 0.40 and 0.59, good for values between 0.60 and 0.74, and excellent for values between 0.75 and 1.0.

There are several ICC forms that could be used that are slightly different from each other. In brief, the different forms of ICC are based on the “Model”, “Type” and “Definition” of the relationship. The “Model” could be a one-way random effects, two-way random effects, or two-way fixed effects or a two-way mixed effects (1-way fixed and 1-way random). The “Type” could be a single rater or the mean of raters and the “Definition” of relationship could be an absolute agreement or consistency [27]. As in our study all included videos (random sample) were analyzed by all three involved research assistants (fixed assessors), a “two-way mixed effect” was used to calculate the ICC. We studied both the reliability in case the T-NOTECHS would be used by a single rater and three raters. Therefore, both “types” of ICCs were calculated. Finally, we calculated both the absolute agreement and consistency ICC (aICC and cICC). Absolute agreement concerns if different raters assign the same score to the same subject. Conversely, relative agreement concerns if different raters assign the same rank ordering of subjects.

### Ethical consideration

The Medical Ethical Committee of the University Medical Center Utrecht has approved the study (reference number WAG/mb/18/022906). Thereby, as agreed with the hospital’s legal department, no informed consent from patient nor personnel needs to be obtained, as our institution makes use of video registration as part of local quality audits. Besides the non-technical skills, no other data of hospital personnel was gathered. Videos of resuscitation were stored on a secured server and all captured videos were analyzed and automatically deleted after 14 days. Thereby T-NOTECHS scores were anonymously stored, which means that a T-NOTECHS score is not traceable to a specific trauma team member or patient.

## Results

### Baseline and assessment scores

Eighteen videos of 18 consecutive trauma team resuscitations were included and assessed by three observers. No resuscitations were missed. The total mean score of the T-NOTECHS was 19 out of 25 graded by all three assessors (Table 1). The domain ‘situation awareness and coping with stress’ had the highest mean score (4.1/5) and the domain ‘leadership’ the lowest mean score (3.6/5) (Table 1). What stands out of Table 2 is that all patients were injured following blunt trauma.

**Table 1:** Mean T-NOTECHS scores

T-NOTECHS domains	Mean score (SD)			
	Rater 1	Rater 2	Rater 3	Mean of raters
Leadership	3.5 (1.0)	3.8 (1.2)	3.3 (0.9)	3.5 (0.9)
Cooperation and resource management	3.8 (0.8)	3.7 (0.8)	3.7 (0.8)	3.7 (0.8)
Communication and interaction	3.6 (1.1)	3.7 (1.0)	3.6 (1.2)	3.6 (1.0)
Assessment and decision making	3.6 (0.9)	3.9 (0.8)	3.7 (0.9)	3.8 (0.8)
Situation awareness and coping with stress	4.4 (0.4)	4.2 (1.0)	4.2 (1.0)	4.1 (0.7)
Overall T-NOTECHS	19 (3.6)	19 (4.5)	19 (4.3)	19 (3.9)

**Table 2:** Baseline characteristics of resuscitated population (P25–P75: 25 and 75th percentile, ISS: Injury Severity Score, GCS: Glasgow Coma Scale)

Baseline characteristics	Observed group (*n* = 18)
Male gender	39%
Median age (P25–P75)	47 (16–66)
Trauma mechanism	
Blunt	100%
Penetrating/other	0.0%
ISS (median, P25–P75)	9.5 (2–14)
Multitrauma patients	33%
Severe TBU patients (GCS ≤ 8)	17%
Deaths	5.6%

### Reliability

The difference for absolute ICC (aICC) and consistency ICC (cICC) for the T-NOTECHS overall score and each domain was small (maximal 0.01). The calculated reliability of the T-NOTECHS was different when the resuscitation was assessed by a single or three raters. When reliability was calculated for the mean of three assessors, the overall score and each domain were ‘excellent’, as the calculated ICC values were between 0.95 and 0.76. The highest reliability was found in the domain ‘Cooperation and resource management’ (aICC = 0.95, 95% CI 0.89–0.98) and the lowest reliability was found in the domain ‘Leadership’ (aICC = 0.76. 95% 0.49–0.90) (Table 3). When reliability was calculated for a single assessor, the reliability was less compared to mean of three assessors (Table 3). Single assessor reliability was ‘good’ for scoring the domains ‘Communication and interaction’ (aICC = 0.73, 95% CI) and ‘Assessment and decision making’ (aICC = 0.59) The scoring domains ‘’Leadership’ (aICC = 0.52) and ‘Situation awareness and coping with stress’ (aICC = 0.59) had a only a ‘fair’ single assessor reliability. The aICC was also lower for the overall T-NOTECHS score and the domain ‘Cooperation and resource management' compared to the reliability when reliability was calculated as a mean of three assessors; however, the score was still ‘excellent’ (aICC = 0.86 resp. 0.84) (Table 3).

**Table 3:** Reliability and performance scores of T-NOTECHS (scores according to Cicchetti et al. [22])

T-NOTECHS domains	Average degree of agreement			Individual degree of agreement		
	Absolute	Consistency	Score	Absolute	Consistency	Score
	ICC (95% CI)	ICC (95% CI)		ICC (95% CI)	ICC (95% CI)	
Leadership	0.76 (0.49–0.90)	0.77 (0.50–0.91)	Excellent	0.52 (0.25–0.76)	0.53 (0.25–0.77)	Fair
Cooperation and resource management	0.95 (0.88–0.98)	0.95 (0.89–0.98)	Excellent	0.86 (0.72–0.94)	0.86 (0.73–0.91)	Excellent
Communication and interaction	0.89 (0.76–0.96)	0.88 (0.75–0.95)	Excellent	0.73 (0.51–0.88)	0.72 (0.50–0.87)	Good
Assessment and decision making	0.90 (0.76–0.96)	0.90 (0.77–0.96)	Excellent	0.73 (0.51–0.87)	0.74 (0.54–0.89)	Good
Situation awareness and coping with stress	0.81 (0.58–0.92)	0.81 (0.57–0.92)	Excellent	0.59 (0.32–0.80)	0.58 (0.31–0.80)	Fair
Overall T-NOTECHS	0.94 (0.87–0.98)	0.94 (0.87–0.98)	Excellent	0.84 (0.70–0.93)	0.84 (0.69–0.93)	Excellent

## Discussion

Our most important finding is that assessment of non-technical skills of the trauma team in real trauma resuscitation using the T-NOTECHS is reliable using video analysis. We found an excellent reliability for the overall T-NOTECHS score. Our second most important finding is that the T-NOTECHS is even more reliable when scores are demonstrated as the mean of three assessors, while all five individual domains instead of two of the T-NOTECHS achieved the highest reliability score. We hope that our research will be helpful in solving the difficulty of measuring non-technical skills during trauma resuscitation. The most important implication of the excellent reliability of the T-NOTECHS tool using video analysis is the possibility to assess the development of non-technical skills over time.

We found a much higher ICC for T-NOTECHS scores than reported by Steinemann et al. [18]. We found an ICC of 0.94 and 0.84, respectively, when measured as the mean of three assessors or a single assessor using video analysis, while in their study an ICC of 0.48 was found for assessment of actual resuscitations by live observers. A possible explanation could be that video analysis instead of live observation may have a positive influence on the reliability of the T-NOTECHS. This suggestion is further supported by results of T-NOTECHS reliability for simulated resuscitation in their study. They found higher T-NOTECHS values using video analysis compared to assessment by live observers (ICC0.44 vs ICC 0.71). In their study, in contrast to this study, no video analysis was used for actual trauma resuscitations, because of hospital policies. Another explanation that our ICC was higher than the study of Steinmann et al. [18] could be a result of our training and experience in trauma resuscitation assessment of the assessors prior to the start of the study.

Overall, other variants of the NOTECHS measuring teamwork during surgery have shown to be reliable. Nevertheless, the results of previous studies investigating the reliability of the NOTECHS are not comparable to our study in exact terms, while different study designs, populations and statistics were used [18, 28, 29]. In the study of Sevdalis et al. [20], the NOTECHS was used by a psychiatrist who observed and assessed non-technical skills among surgical teams in a simulated setting. In this study, the reliability was calculated using Cronbach's alpha (*α*) internal consistency coefficients, which provide the same values as a two-way consistency ICC of average measurements (in our study a two-way mixed ICC was used) and, therefore, not completely, but most comparable to our mean ICC results [20, 30]. The NOTECHS tool used in their study had also five domains, which are comparable to t T-NOTECHS, but adjusted for surgical team performance. Like the T-NOTECHS, the NOTECHS in their study had a five-point Likert-scale for each five individual domain. The most reliable domain had a Cronbach’s *α* of 0.87 and the least reliable domain had a score of 0.77. In the study of Mishra et al. [28], a single observer assessed non-technical skills of individual team members, subteams and the team as a whole using the Oxford NOTECHS. The Oxford NOTECHS is comparable to T-NOTECHS in number and sort domains, but adjusted for surgical team assessment. Thereby, domains were scored on a four-point Likert-scale for each member and points were summed up for each subteam (4–16 points) and overall team score (12–48). Reliability was tested using inter-rater agreement (Rwg). The overall NOTECHS Rwg for the team was 0.99. and the lowest domain for the team had an overall score of Rwg 0.93. These high scores indicate that the tool is very reliable; however, using Rwg to assess reliability in their study design may have introduced analytical bias. Analyses by Rwg uses a null hypothesis of complete lack of agreement among raters, which is in their study means that all of the 37 options for overall team score (all possible outcomes when individual scores are summed up) had an equal chance (i.e., 1/37 or 2,7%) of being scored by the assessor. Such a distribution is very unlikely, which was more or less confirmed by the statement in the article of Robertson et al. [29] presenting the successor of the Oxford NOTECHS, the Oxford NOTECHS II. The authors wrote that the successor intended to provide greater discrimination, as teams scored within a narrow middle range in the first Oxford NOTECHS version. The Oxford NOTECHS II had the same fundamentals compared to the Oxford NOTECHS, but the scale was altered. Reliability of the Oxford NOTECHS II was measured using ICC, without description of what kind of ICC model, type or definition was used and therefore no proper comparison to our results could be made. The ICC for the individual domains was between 0.68 and 0.88.

Although our sample size was intuitively small, our study design included a sample size calculation and our study was able to adequately indicate the reliability of T-NOTECHS for a single and multiple assessor by video analyses. Another strength of this study is that real trauma resuscitation was analyzed (instead of simulations). However, our study has also several limitations that should be considered. First, we were not able to properly assess intra-observer variability. Videos of trauma resuscitations are automatically deleted from the server after 30 days, because of local hospital’s security and privacy policies. Assessing the same video within 2 weeks would have introduced recall bias. Second, in this study we assessed non-technical skills of the trauma team during resuscitations. The trauma team is activated for potentially severely injured patients, which is predefined by anatomical, physiological criteria or mechanism of trauma; however, the mean ISS of resuscitated patients in this study was relatively low [9]. Therefore, our results may be less representative for resuscitations of more severely injured patients. Third, we used a two-way mixed-effects, as only three research assistants were used to assess non-technical skills. We have chosen to assess non-technical skills by adequately trained personnel with the intention to improve the validity and reliability of our measurements. The downside of choosing a limited number of trained personnel is, in exact terms, that we tested the reliability of non-technical skills assessment of our trained research assistants. Therefore, caution should be exercised when generalizing our results, while our results might overestimate the reliability of T-NOTECHS. Finally, our assessors were trained medical students, which intuitively might be inferior to assessment by experienced clinical experts. However, these students had already had training and gained experience in the assessment of trauma resuscitation and had extensive training in the assessment of non-technical skills. To our knowledge, for trauma resuscitations specifically, no study has investigated the effect of raters’ education on the reliability of non-technical skills assessment of trauma teams. Nevertheless, a considerable amount of literature has been published on the use of objective structured clinical examinations (OSCEs), which have become widely used in medical education [31]. Medical schools have invested significant resources in designing and implementing OSCE in assessment programs, with the rigor of the process highly dependent on whether OSCEs provide reliable and valid indicators of student competence [32] Research suggests that untrained raters may be less consistent than trained raters [33, 34]. In addition, raters with more clinical experience are not naturally better assessors of non-technical skills. A recently published study of Pradarelli et al. [35] showed that clinical experience of raters, in their study surgeons, had no effect on reliability of non-technical skill assessment of other surgeons. Furthermore, from a practical viewpoint, routine assessment of resuscitation is very time consuming and, in our opinion, not feasible to be performed in the precious time of experienced clinicians. Overall, assessment by other personnel than experienced clinicians is more likely to be incorporated in daily practice. Therefore, we believe that the reliability we found is appropriate for the purpose of T-NOTECHS.

As evidence supporting the importance of non-technical skill for trauma team resuscitation is growing rapidly [6–17], training of non-technical skills becomes more important. For instance, closed loop communication has shown to reduce overall resuscitation time [36]. Furthermore, enhanced leadership is positively associated with improvement of processes during resuscitation [37]. The T-NOTECHS might be a useful, and to our knowledge, best available tool to assess non-technical skills of the trauma team. For daily practice, one rater to assess non-technical skills using the T-NOTECHS seems legitimate as part of quality assessment, while the overall score is a reliable value. For research or quality improvement, it might be interesting to secondarily assess non-technical skills with three raters. For example, when (relatively) low overall T-NOTECHS scores are correlated to a certain factor (e.g., trauma mechanism, severity of injury, experience of trauma team), an analysis with three raters would be useful.

## Conclusion

Assessment of non-technical skills using the T-NOTECHS is reliable using video analysis and has an excellent reliability for the overall T-NOTECHS score. Assessment by three raters (score as a mean) further improves the reliability, resulting in an excellent reliability for all individual domains.
